# Events of alternative splicing in head and neck cancer via RNA sequencing – an update

**DOI:** 10.1186/s12864-019-5794-y

**Published:** 2019-06-03

**Authors:** Vishwas Sharma, Amrita Nandan, Harpreet Singh, Suyash Agarwal, Richa Tripathi, Dhirendra Narain Sinha, Ravi Mehrotra

**Affiliations:** 1 Department of Health Research, National Institute of Cancer Prevention and Research, Noida, Uttar Pradesh India; 2 Society for Life Science and Human Health, Allahabad, Uttar Pradesh India; 3 ICMR Computational Genomics Centre, Indian Council of Medical Research, New Delhi, 110029 India; 4 Informatics, Systems and Research Management, Indian Council of Medical Research, New Delhi, 110029 India; 5 Division of Molecular Cytology, National Institute of Cancer Prevention and Research, Noida, Uttar Pradesh India; 6 WHO FCTC Global Knowledge Hub on Smokeless Tobacco, National Institute of Cancer Prevention and Research, Noida, Uttar Pradesh India

**Keywords:** Alternative splicing, Exon skipping, Head and neck cancer, Mutually exclusive exon, Retained introns, RNA-Seq, Transcriptome

## Abstract

**Background:**

*Alternative splicing* (AS) is a regulatory mechanism used to create many forms of mature messengers RNAs (mRNAs) from the same gene. Sequencing of RNA (RNA-Seq) is an advanced technology, which has been utilized by different studies to find AS mechanisms in head and neck cancer (HNC). Hitherto, there is no available review that could inform us of the major findings from these studies. Hence, we aim to perform a systematic literature search following PRISMA guidelines to study AS events in HNC identified through RNA-Seq studies.

**Results:**

A total of five records were identified that utilized RNA-Seq data for identifying AS events in HNC. Five software was used in these records to identify AS events. Two genes influenced by AS i.e. *MLL3* and *RPS9* were found to be common in 4 out of 5 records. Likewise, 38 genes were identified to be similar in at least 3 records.

**Conclusions:**

Alternative splicing in HNC is a multifaceted regulatory mechanism of gene expression. It can be studied via RNA-Seq using different bioinformatics tools. Genes *MLL3*, as well as RPS9, were repeatedly found to be associated with HNC, however needs further functional validation.

**Electronic supplementary material:**

The online version of this article (10.1186/s12864-019-5794-y) contains supplementary material, which is available to authorized users.

## Background

Alternative splicing (AS) is a key molecular mechanism through which single gene gives rise to multiple differently spliced forms of a protein [1], hence provides greater diversity at transcriptomics and proteomics level [2]. The four major subtypes of AS include a) cassette exon skipping (ES): results in the loss of an exon together with its flanking introns in the alternatively spliced mRNA [3], b) intron retention (IR): part of an exon is either spliced (similar to intron) or retained in the mature mRNA [4–7], c) mutually exclusive exon (MXE): exactly one out of two exons (or one group out of two exon groups) is retained, while the other is spliced out [6] and d) alternative 5’and 3′ splice site (ASS): two or more alternative 5′ splice site competes for joining with two or greater than two alternate 3′ splice site [8] (details on Additional files 1 and 2Figure S1). Alternative splicing may result in the addition or elimination of a stop codon in the alternative coding sequence or use of a different translation termination/ initiation site due to a frameshift. Alternative splicing may also result in a change in the internal region of a gene due to an in-frame insertion or deletion [9].

Classically real-time RT-PCR or array-based methods were used to detect AS events, but had their own limitations as they required the prior knowledge of the genome sequence [10, 11]. This shortcoming has been overcome by modern sequencing of RNA (RNA-Seq) technology, which does not require the existing knowledge of genome sequence, and hence could be crucial in identifying previously unidentified genes and splicing events [5]. This technology has expedited the process of identifying all four AS subtypes in cancers [12] and has also been implemented to study the mechanism/s of AS in head and neck cancer (HNC) [13], the sixth most common cancer in men, comprising around 6% of all cancer incidents [14]. Till date, there is no available review of the literature (Additional file 3) that could update us with the recent developments in finding AS events in HNC via RNA-Seq data. Absence of any relevant literature inspired us to perform a comprehensive review with the aim to highlight the importance of studying AS events in HNC captured through RNA-Seq data for identifying molecular clues for future research and therapy in HNC.

## Methods

**Fig. 1:**
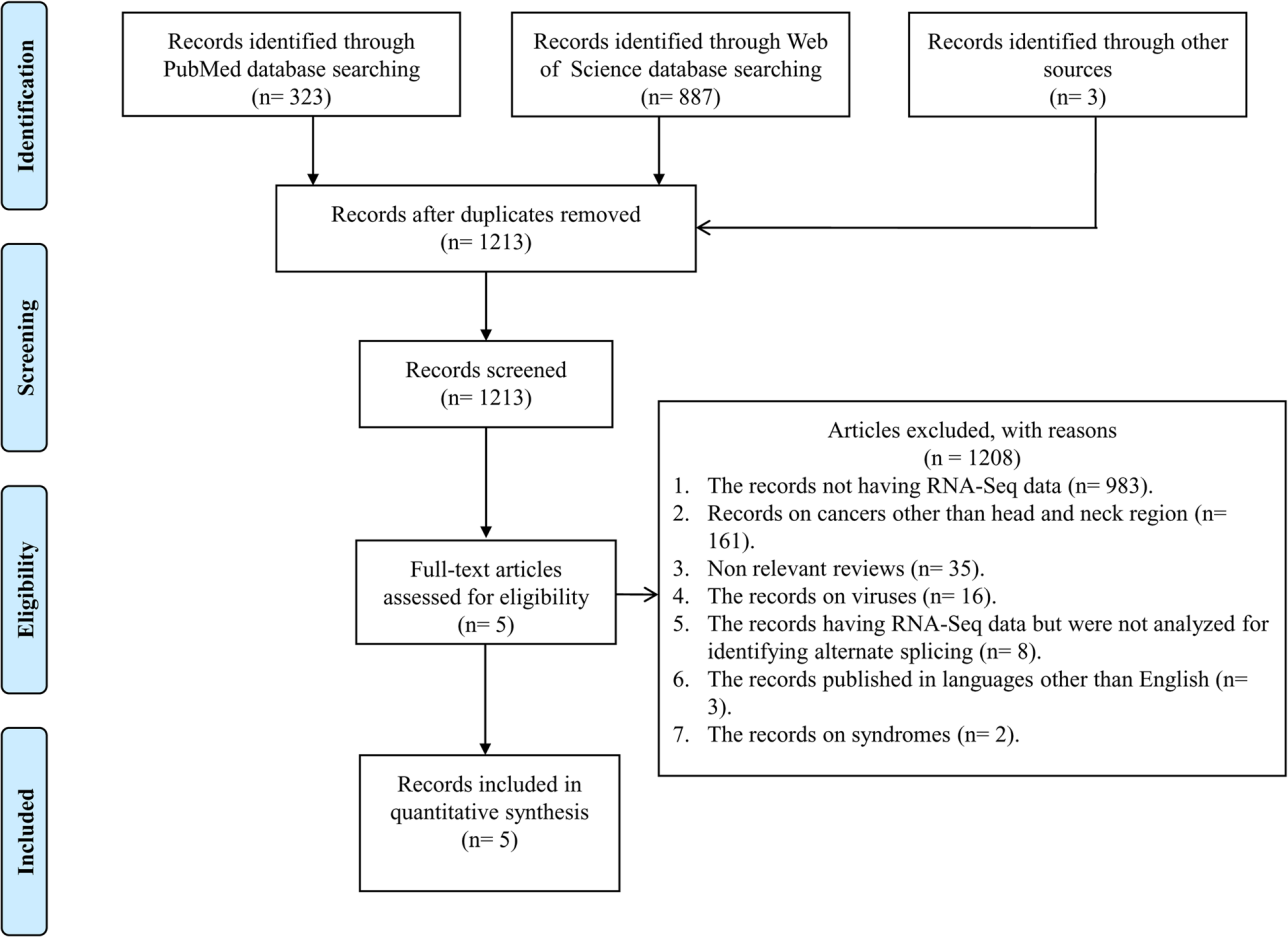
Flow chart describing the included/excluded literature

A comprehensive search was performed to know the AS events in HNC identified through RNA- Seq (Fig. 1 and Additional files 3 and 4Table S1). Briefly, records were screened on PubMed and Web of Science databases on the 19th of September, 2017. In addition, Google (1st November 2017) was also consulted for including records not captured through PubMed and Web of Science search. A total of 323 records were obtained from PubMed, 887 records from Web of Science and 3 additional from Google search. None of the records duplicated from databases, hence a total of 1213 records were obtained during a comprehensive search. Similar phrases were used for searching articles in PubMed and Web of Science. The only difference is with Web of Science where records published between the years 2010 to 2017 were considered. The details of phrases used for searching records are mentioned in Additional file 3.

The records were screened using inclusion and exclusion criteria mentioned below-.

### Inclusion criteria

Original studies that utilized RNA-Seq data for identifying AS events in human oral cancer (OC), oral squamous cell carcinoma (OSCC), squamous cell carcinoma of the oral cavity, squamous cell carcinoma of lip, squamous cell carcinoma of buccal mucosa, squamous cell carcinoma of tongue, carcinoma of floor of the mouth, carcinoma of palate, carcinoma of gingival, carcinoma of maxillary sinus, verrucous carcinoma, Lane tumor, adenoid cystic carcinoma, salivary duct carcinoma, salivary duct adenocarcinoma, ghost cell odontogenic carcinoma, clear cell odontogenic carcinoma, cancers of the nasal cavity, nasopharyngeal carcinoma, African jaw lymphoma, oropharyngeal cancer, laryngeal cancer, laryngeal squamous cell carcinoma, hypopharyngeal cancer, head and neck cancer, head and neck squamous cell carcinoma (HNSCC) were considered {*n* = 5; identified records comprises oropharyngeal squamous cell carcinoma (OPSCC), OC, buccal mucosa squamous cell carcinoma (BMSCC) and other subtypes of HNC} (Additional file 4 Table S1).

### Exclusion criteria

- The records not having RNA-Seq data (*n* = 983).
- Records on cancers other than head and neck region (*n* = 161).
- Non relevant reviews (*n* = 35).
- The records on viruses (n = 16).
- The records having RNA-Seq data but were not analyzed for identifying alternative splicing (*n* = 8).
- The records published in languages other than English (n = 3).
- The records on syndromes (*n* = 2).

### Filtering common genes having AS events

**Fig. 2:**
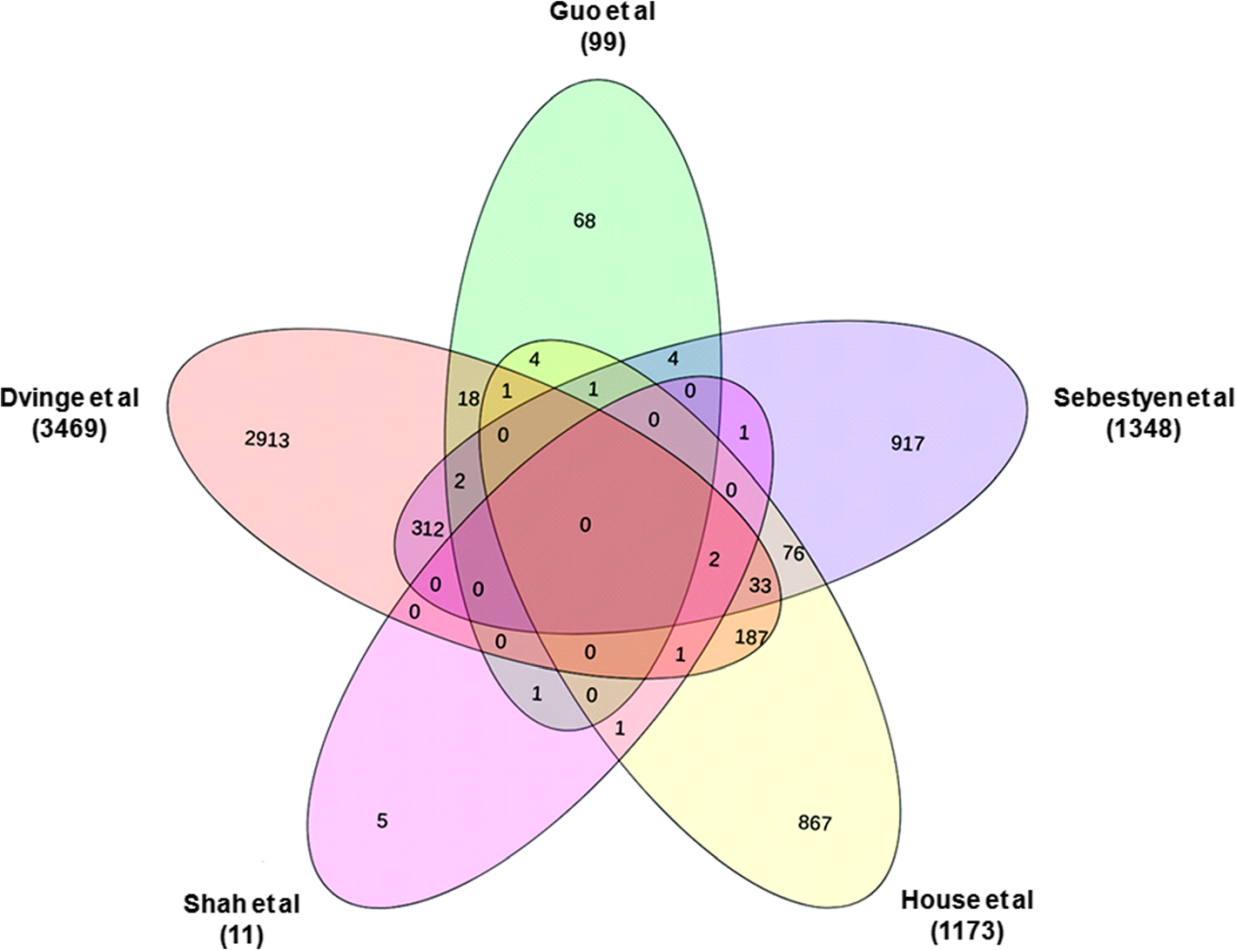
Venn diagram showing the comparative genes analysis among 5 records

In order to find out common genes showing AS events in at least 3 out of 5 independent records, the gene list of all the 5 records was compared (Fig. 2 and Table 1).

**Table 1:** Details of genes common in three or four records

	Total genes	Studies under consideration	Total number of common genes	Genes
Genes common in four studies	2	Dvinge *et*. [15], Sebestyén et al. [17], House et al. [13] and Shah et al. [18]	2	*MLL3*, *RPS9*
Genes common in three studies	38	Dvinge et al. [15], Guo et al. [16] and Sebestyén et al. [17]	2	*CLK1*, *CLK4*
		Dvinge et al. [15], Guo et al. [16] and House et al. [13]	1	*BAI2*
		Guo et al. [16], Sebestyén et al. [17] and House et al. [13]	1	*S100A4*
		Dvinge et al. [15], Sebestyén et al. [17] and House et al. [13]	33	*ATXN2L*, *BCLAF1*, *C1orf35*, *CCDC12*, *DIAPH1*, *EIF4G1*, *FXR1*, *HDLBP*, *HMG20B*, *HSPB1*, *INTS6*, *LAS1L*, *NSUN5*, *PCBP2*, *PRPF31*, *PRPF40B*, *RBM8A*, *RPS11*, *RPS18*, *RPS20*, *RPS25*, *RPS26*, *RPS27A*, *RPS28*, *SLC16A3*, *SLC25A11*, *SPTAN1*, *TAGLN2*, *TBCA*, *TSEN2*, *U2AF1*, *WBSCR22*, *YTHDF3*
		Dvinge et al. [15], House et al. [13] and Shah et al. [18]	1	*STAT6*

### Network and functional enrichment analysis

The network and functional enrichment analysis of all the genes, which were common in either 3 or 4 records was done using Cytoscape (version 3.7.1) which uses the STRING database as a data source. Gene ontology and pathway enrichment analysis was carried out at FDR value <= 0.05. The Cytoscape plugin, cytoHubba was used in order to explore the hub genes from the interactome network, based on eleven topological algorithms including Degree, Edge Percolated Component (EPC), Maximum Neighborhood Component (MNC), Density of Maximum Neighborhood Component (DMNC), Maximal Clique Centrality (MCC) and centralities based on shortest paths as Bottleneck (BN), EcCentricity, Closeness, Radiality, Betweenness, and Stress.

## Results

Five records were identified that implemented RNA-Seq data for finding AS events in HNC (Fig. 1). (i) A recent study by Guo et al. [16] conducted on 46 HPV-positive OPSCC and 25 normal control subjects, characterized 109 splicing events on the basis of junctions location and configuration. The junctions were further characterized as ASS (39%), insertion (21%), canonical exon skipping (9%), intron retention (9%), deletion (8%), alternate end site (7%), or noncoding (3%). Of the total 109 validated splicing events, 70% were identified as noncanonical splicing events. Since the noncanonical splicing event contributes to 1% of the total splicing events in normal cells, the abundance of this phenomenon in cancerous cells indicates its importance for further research. Data analysis through MapSplice software [23] resulted in the identification of functionally active splice variant of *AKT3* in HPV positive OPSCC primary tumors. The AKT is a signal transduction pathway involved in cell growth and survival in response to extracellular signals and is well known to be linked with the pathogenesis of HNSCC [16]. The observed correlation between DNA methylation and alternative start site of AS events in this study are suggested to be important for further investigation. By utilizing a unique method of analysis the authors filtered 100 tumor-specific splice variant candidates in HPV positive HNSCC. The method described in this study could be followed for the identification of novel splicing events and to better understand the complex perturbations occurring in the tumor cells. Parallel identification of correlated methylation patterns could add in defining targets for validation and therapy. Comparison of AS events between different tumor types may give a clue towards a defined structure of module followed by either the tumor cells collectively or a given group of tumor types based on origin. This module could be further validated and targeted to define a treatment regime. (ii) Studying AS events by using a computational tool SUPPA [19], Sebestyén, et al. [17] identified RNA binding motif protein 47 (*RBM47*) tumor suppressor gene to be the main common splicing factor between kidney renal papillary carcinoma and HNSCC. The study reported 141 common AS events between HNSCC and Lung Squamous Cell Carcinoma and 63 out of the 141 splicing events to be associated with RNA binding motif protein 28 (*RBM28*). The analysis was performed on 38 HNSCC and 38 control sample data downloaded from the TCGA data portal [32]. The study reported the importance of alterations in the genes encoding RNA binding proteins and that they trigger common and specific AS events in various solid tumors. Thus, suggesting a pattern of AS events followed by several solid tumors that could be studied to understand the molecular basis and oncogenic properties of tumor cells for defining treatment modalities. (iii) An IR has been recently observed to be an important factor behind the disruption of normal splicing machinery in cancerous cells [15, 33]. An important finding that abnormal RNA splicing, characterized by widespread IR events, is common across cancers was marked by a study performed by Dvinge and Bradley [15] on 40 HNC patients data downloaded from CGHub [34]. The mechanism of IR is a multifactorial process and it is suggested that transcriptional diversity in cancer cells may be driven by at least some intron-containing mRNAs that are adequately stable [15]. Whether these introns containing mRNAs are translated into proteins or degraded still remains an interesting topic of investigation. Studying AS events identified 829 cassette exons, 153 competing 5′ splice sites, 202 competing 3′ splice sites, 115 retained introns, 290 constitutive introns and 418 constitutive junctions. Moreover, crosstalk between RNA splicing events and epigenetics may be associated with a diverse spectrum of mutations to RNA processing defects in cancer. The bioinformatic tool MISO [21] which is based on statistical models that estimate expression of alternatively spliced exons and isoforms and evaluates confidence in these estimates was applied for the RNA-Seq data analysis in the study. (iv) To investigate the role of CUGBP embryonic lethal abnormal vision-like family member 1 (CELF1), an important regulator of AS, editing and translation, in OC, House et al. [13] used RNA-Seq data to analyze AS variants in sicontrol and siCELF1 transfected OC cells. The AltAnalyze software which runs on two algorithms to quantify AS events, i.e. ASPIRE (Analysis of Splicing Isoform Reciprocity) and splicing index was utilized to perform the analysis [20]. The former finds AS events by matching-up the levels of the alternatively expressed exons and exon-exon junctions whereas the latter derives AS events via normalizing single exon expression to total gene expression levels. The study captured 315 AS events comprising 282 genes. Cell cycle, microtubule-based processes and translocation initiation constituted the three most significant over-represented biological functions of 282 splicing targets. The most common AS events regulated by CELF1 were ES and inclusion. CELF1 is a critical regulator of cancer cell proliferation and apoptosis. Since the authors were interested in studying CELF1 mediated AS events in three pre-mRNAs (COL16A1, TACC2 and ITGA6) known to play a role in cell growth, they found a modest difference in the expression for *COL16A1*, *TACC2*, and *ITGA6* CELF1-mediated splicing events in HNSCC tumors in comparison to normal samples. Therefore, the rest of the data still remains to be studied for other mechanisms influenced by deregulation of CELF1 in HNSCC. (v) While analyzing data from BMSCC through SpliceMap software [22] Shah et al. [18] found 11 novel splice junctions, encompassing *IMPA2*, *CD44*, *FAM48A*, *DHX32*, *MLL3*, *STAT6*, *FAM82B*, *SPICE1*, *MEMO1*, *RPS9* and *SPINK5* genes, mostly derived from 5′ ASS. All of them were known to alter the coding region and cause an alteration in protein sequence leading to gain or loss of protein function and change in specificity. Thus a relevant biological consequence of AS events was reported through this study. All the studies under consideration showed the IR event as the major component of AS in HNC, with the only exception being the study by Shah et al where IR was not detected as an AS event. This discrepancy could be due to the difference in the sequencing platform used, as others [13, 15–17] utilized Illumina however Shah et al. [18] used Roche 454 to generate the RNA-Seq data. Moreover, most of the novel splice junctions reported in the study were supported either by two reads or a single read. The AS events supported by a single read were excluded from further analysis which could be possible IR events and may have been captured if the sequencing depth was more.

Two genes, i.e. *MLL3* and *RPS9* were found to be common in 4 out of 5 records (Table 1 and Fig. 2). Similarly, 38 genes were identified to be common in 3 records (Table 1). Hence, a total of 40 genes were used for gene enrichment analysis. In the records identified, the authors analyzed the RNA-Seq data using five softwares i.e. Suppa [19], AltAnalyze [20], MISO (Mixture of Isoforms Probablistic Model) [21] SpliceMap [22] and MapSplice [23], for finding AS events in HNC. The top 10 gene ontology enrichment pertaining to the cellular component, molecular function, and biological process are depicted in Additional file 5: Figure S2. The major pathways being regulated by these genes include ribosome, spliceosome and RNA transport (Additional file 6). The protein-protein network of all the 40 genes was (Additional file 7Figure S3) analyzed using cytoHubba resulting in 5 genes which passed more than 10 topological algorithms, out of eleven to be called as the major HUB genes which include *RPS28*, *RBM8A*, *RPS9*, *EIF4G1* and *RPS27A* (Additional file 6).

## Discussion

Alternative splicing of genes is believed to be associated with the initiation, progression, and metastasis of cancer [24–26]. Differences in RNA processing may differentiate cancer and normal cells, as spliceosomal components are differentially required in cancer versus normal cells [27, 28]. Due to these differences, recent ongoing research on antitumor drugs focuses on the inhibitors of RNA splicing [29–31]. In the last few years, clinicians/researchers have utilized the power of RNA-Seq for understanding the mechanism/s of AS at genome-wide level, that was not feasible previously with classical methods. Knowledge of these events will assist us in understanding the complex regulation of gene expression in cancer. Recently, an abundance of effort has been put into extracting information about AS in HNC via RNA-Seq data, with the aim to utilize/implement this information in finding new drug targets or searching candidate molecular markers for early and accurate diagnosis of the disease. Considering the significance of the topic, the literature was reviewed and compiled to provide information on the recent ongoing in finding AS events, through RNA-Seq data, for HNC.

The subsequent literature search led to the filtration of five records associated with RNA-Seq data analysis for discovering AS events in HNC. Four records identified *MLL3* and *RPS9* as a common gene influenced by AS in HNSCC [13, 15, 17, 18]. The *MLL3* (mixed lineage leukemia-3) encodes histone-modifying enzyme characterized by a conserved catalytic SET domain and is involved in transcriptional coactivation. *MLL3* is considered as a tumor suppressor gene because of its frequent mutations in several tumor types [35, 36]. Recurrent mutations in *MLL3* have been reported in HNC in addition to several other cancers, including pancreatic, breast and gastric [37–41]. In a study by Mountzios et al, [37] analysis of the data from “The Cancer Genome Atlas”, *MLL3* was identified with a mutation rate of 7.3% in HNC [37]. Downregulated expression of *MLL3* is reported to contribute to tumor progression in HNSCC [42]. Additionally, reduced expression of *MLL3* is reported in breast tumors and gastric cancer [43, 44]. Ribosomal Protein S9 (*RPS9*) is one of the first proteins that directly bind to the 18S rRNA and plays a crucial role in ribosome biogenesis [45–47]. Inhibition of RPS9 activates p53 activity, it is suggested to be an efficient method to induce apoptosis in tumor cells [48]. Several studies have reported the role of RPS9 in tumorigenesis [45, 48]. Since the protein induces the p53 pathway and was observed to be involved in HNC, futuristic validation is required for its functional importance in HNC. We have discussed genes common in 4 records, however additional 38 genes common in at least 3 records may also be of biological relevance. The records studied used Suppa [19], AltAnalyze [20], MISO [21] SpliceMap [22] and MapSplice [23], for finding AS events in HNC. Apart from that, there are also other AS detection software as listed in Additional file 8Table S2 which are already implemented in cancer research and hence could be used for finding AS in HNC.

## Conclusion

The comprehensive literature search has indicated towards the gap in utilizing RNA-Seq data to identify and study AS events in HNC. Utilization of RNA-Seq data to study the AS events in HNC is crucial to understand the complex cascade of events regulating gene expression. However, in the current review only 5 articles relevant to AS events in HNC were identified, of which 3 were from patients with HNC, oral cancer, and buccal mucosa tumors, respectively, one study deals with TCGA data and the last has been conducted using cell lines. Although the data is heterogeneous, but due to the limited number of studies available, HNC was considered as an umbrella under which other subtypes such as oral cancer, and buccal mucosa tumor falls. For a more robust interpretation of genes involved in AS events in HNC, a larger number of studies at a subtype level are required from different geographical regions. Interestingly, in next-generation sequencing (NGS) based data repository such as GEO [49], SRA [50], TCGA [32], etc. RNA-Seq data of more than a hundred fifty HNC patients are available and could be analyzed through various bioinformatics softwares. We suggest comparing the data captured on the similar sequencing platform, and analyze them through multiple AS events identification software, followed by the selection of common candidate AS events. With the reducing cost of RNA-Seq technology, it is expected that in the next few years data repositories will be enriched with HNC patient’s data that will help diagnostic/pharma world in finding new therapeutic and molecular targets for detecting the disease at a preliminary stage. This will also assist clinicians in deciding their treatment and management strategies against HNC. Although RNA-Seq data has few limitations [51] but still it is a reliable biological tool for finding novel AS events. In the near future, RNA-Seq technology is expected to be a hallmark in investigating novel AS events for HNC or any other types of carcinoma.

## Additional files

Additional file 1: Alternative splicing (AS) subtypes. (DOC 89 kb)

Additional file 2: **Figure S1.** Alternative splicing subtypes. The figure was adopted and modified from Shen et al. [52]. (TIF 501 kb)

Additional file 3: Criteria of screening studies. (DOC 48 kb)

Additional file 4: **Table S1.** describing the reason for including or excluding each study. (XLS 232 kb)

Additional file 5: **Figure S2.** Gene ontology enrichment graph. (TIFF 206 kb)

Additional file 6: Functional enrichment and hub genes. (XLS 47 kb)

Additional file 7: **Figure S3.** Network analysis of all the 40 common genes. (TIFF 3427 kb)

Additional file 8: **Table S2.** Summary of the alternative splicing detection softwares and their implementation in cancer research. (DOC 139 kb)

## Abbreviations

AS: alternative splicing
ASPIRE: Analysis of Splicing Isoform Reciprocity
ASS: alternative 5’and 3′ splice site
BMSCC: buccal mucosa squamous cell carcinoma
CELF1: CUGBP embryonic lethal abnormal vision-like family member 1
CGHub: Cancer Genomics Hub
ES: exon skipping
GEO: Gene Expression Omnibus
HNC: head and neck cancer
HNSCC: head and neck squamous cell carcinoma
HPV: human papillomavirus
IR: intron retention
MISO: Mixture of Isoforms probablistic model
MLL3: mixed lineage leukemia-3
mRNA: messenger RNA
MXE: mutually exclusive exon
OC: oral cancer
OPSCC: oropharyngeal squamous cell carcinoma
OSCC: oral squamous cell carcinoma
RBM28: RNA binding motif protein 28
RBM47: RNA binding motif protein 47
RNA-Seq: Sequencing of RNA
RPS9: Ribosomal Protein S9
RT-PCR: Real Time-Polymerase Chain Reaction
SRA: Sequence Read Archive
TCGA: The Cancer Genome Atlas
