# Supplementary material for: Events of alternative splicing in head and neck cancer via RNA sequencing – an update
Source: BMC Genomics. 2019 Jun 3;20:442. doi: 10.1186/s12864-019-5794-y (PMC6545735; doi:10.1186/s12864-019-5794-y)
Supplement: Supplementary file 1 — Alternative splicing (AS) subtypes. (DOC 89 kb) [file 12864_2019_5794_MOESM1_ESM.doc]

**Additional file 1**

**Alternate splicing (AS) subtypes**

1. *Cassette exon skipping (ES) subtype*

In this, exon is lost in the alternatively spliced mRNA (Additional file 2). It is the most common mechanism of AS in mammals [1]. These are shown to be frequently present in different types of cancers such as human breast [2], lung [3] prostrate and head and neck cancers (HNC) [4]. Classically, it was detected through array based methods or through real time RT-PCR and sequencing. Using these classical methods i.e TissueScan qPCR arrays, cells skipping of exon 11 of E-cadherin RNA were reported in tumor tissues that downregulates its expression in HNC [4]. Skipping of exons was also identified, through real time RT-PCR and sequencing in the inhibitor of growth 5 (ING5) gene in RNA samples obtained from oral cancer patients that resulted in the formation of five AS variants [5].

1. *Intron retention (IR) subtype*

Here intron is transcribed into pre-mRNA and remains as such in the final mRNA [6] (Additional file 2). It is the rarest subtype of AS in mammals. Increased IR was found to be linked with *SETD2* mutations in the cancer of kidney [7] and metastatic castration resistance in prostate cancer [8]. Moreover, partial retention of intron 6 of *GSTP1* was also identified in a study on HNC patients [9].

1. *Mutually exclusive exon (MXE) subtype*

As the name indicates, one out of two exons or one group out of two exon groups is retained, while the other one is spliced out [10] (Additional file 2). It represents a less abundant subtype of AS events. Studies found that missense mutations in MXEs can lead to diseases in humans [11,12], however its involvement in HNC is still unclear. Plausibly in the upcoming years we may know the role of MXE in HNC development which may help us to develop a comprehensive database of MXE and other AS events in HNC.

1. *ASS subtype*

ASS comprises ~18% and ~8% of the human–mouse conserved events, respectively. These exons are flanked on one side by a constitutive splice site (fixated) and on another side by two or more than two competing alternative splice sites, resulting in an alternative region (extension) which is either included in the transcript or is excluded (Additional file 2). It relates to several aberrant splicing diseases [13].

**References**

1. Wang J, Ye Z, Huang TH, Shi H, Jin VX. Computational Methods and Correlation of Exon-skipping Events with Splicing, Transcription, and Epigenetic Factors. Methods Mol Biol [Internet]. 2017;1513:163–70. Available from: http://www.ncbi.nlm.nih.gov/pubmed/27807836

2. Eswaran J, Horvath A, Godbole S, Reddy SD, Mudvari P, Ohshiro K, et al. RNA sequencing of cancer reveals novel splicing alterations. Sci Rep [Internet]. 2013;3:1689. Available from: http://www.ncbi.nlm.nih.gov/pubmed/23604310

3. Dhanasekaran SM, Balbin OA, Chen G, Nadal E, Kalyana-Sundaram S, Pan J, et al. Transcriptome meta-analysis of lung cancer reveals recurrent aberrations in NRG1 and Hippo pathway genes. Nat Commun [Internet]. 2014;5:5893. Available from: http://www.ncbi.nlm.nih.gov/pubmed/25531467

4. Sharma S, Liao W, Zhou X, Wong DTW, Lichtenstein A. Exon 11 skipping of E-cadherin RNA downregulates its expression in head and neck cancer cells. Mol Cancer Ther [Internet]. 2011;10:1751–9. Available from: http://www.ncbi.nlm.nih.gov/pubmed/21764905

5. Cengiz B, Gunduz E, Gunduz M, Beder LB, Tamamura R, Bagci C, et al. Tumor-specific mutation and downregulation of ING5 detected in oral squamous cell carcinoma. Int J cancer [Internet]. 2010;127:2088–94. Available from: http://www.ncbi.nlm.nih.gov/pubmed/20131318

6. Middleton R, Gao D, Thomas A, Singh B, Au A, Wong JJ-L, et al. IRFinder: assessing the impact of intron retention on mammalian gene expression. Genome Biol [Internet]. 2017;18:51. Available from: http://www.ncbi.nlm.nih.gov/pubmed/28298237

7. Simon JM, Hacker KE, Singh D, Brannon AR, Parker JS, Weiser M, et al. Variation in chromatin accessibility in human kidney cancer links H3K36 methyltransferase loss with widespread RNA processing defects. Genome Res [Internet]. 2014;24:241–50. Available from: http://www.ncbi.nlm.nih.gov/pubmed/24158655

8. Sowalsky AG, Xia Z, Wang L, Zhao H, Chen S, Bubley GJ, et al. Whole transcriptome sequencing reveals extensive unspliced mRNA in metastatic castration-resistant prostate cancer. Mol Cancer Res [Internet]. 2015;13:98–106. Available from: http://www.ncbi.nlm.nih.gov/pubmed/25189356

9. Masood N, Malik FA, Kayani MA. Unusual intronic variant in GSTP1 in head and neck cancer in Pakistan. Asian Pac J Cancer Prev [Internet]. 2012;13:1683–6. Available from: http://www.ncbi.nlm.nih.gov/pubmed/22799388

10. Sammeth M. Complete alternative splicing events are bubbles in splicing graphs. J Comput Biol [Internet]. 2009;16:1117–40. Available from: http://www.ncbi.nlm.nih.gov/pubmed/19689216

11. Mayr JA, Zimmermann FA, Horváth R, Schneider H-C, Schoser B, Holinski-Feder E, et al. Deficiency of the mitochondrial phosphate carrier presenting as myopathy and cardiomyopathy in a family with three affected children. Neuromuscul Disord [Internet]. 2011;21:803–8. Available from: http://www.ncbi.nlm.nih.gov/pubmed/21763135

12. Tang ZZ, Sharma S, Zheng S, Chawla G, Nikolic J, Black DL. Regulation of the mutually exclusive exons 8a and 8 in the CaV1.2 calcium channel transcript by polypyrimidine tract-binding protein. J Biol Chem [Internet]. 2011;286:10007–16. Available from: http://www.ncbi.nlm.nih.gov/pubmed/21282112

13. Koren E, Lev-Maor G, Ast G. The emergence of alternative 3’ and 5’ splice site exons from constitutive exons. PLoS Comput Biol [Internet]. 2007;3:e95. Available from: http://www.ncbi.nlm.nih.gov/pubmed/17530917
