# Supplementary figures and images for: Events of alternative splicing in head and neck cancer via RNA sequencing – an update
Source: BMC Genomics. 2019 Jun 3;20:442. doi: 10.1186/s12864-019-5794-y (PMC6545735; doi:10.1186/s12864-019-5794-y)

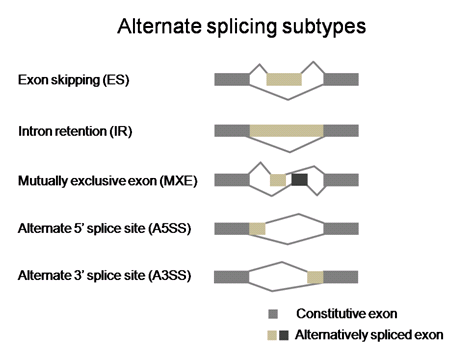

Supplement: Supplementary file 2 — Figure S1. Alternative splicing subtypes. The figure was adopted and modified from Shen et al. [52]. (TIF 501 kb) [file 12864_2019_5794_MOESM2_ESM.tif]

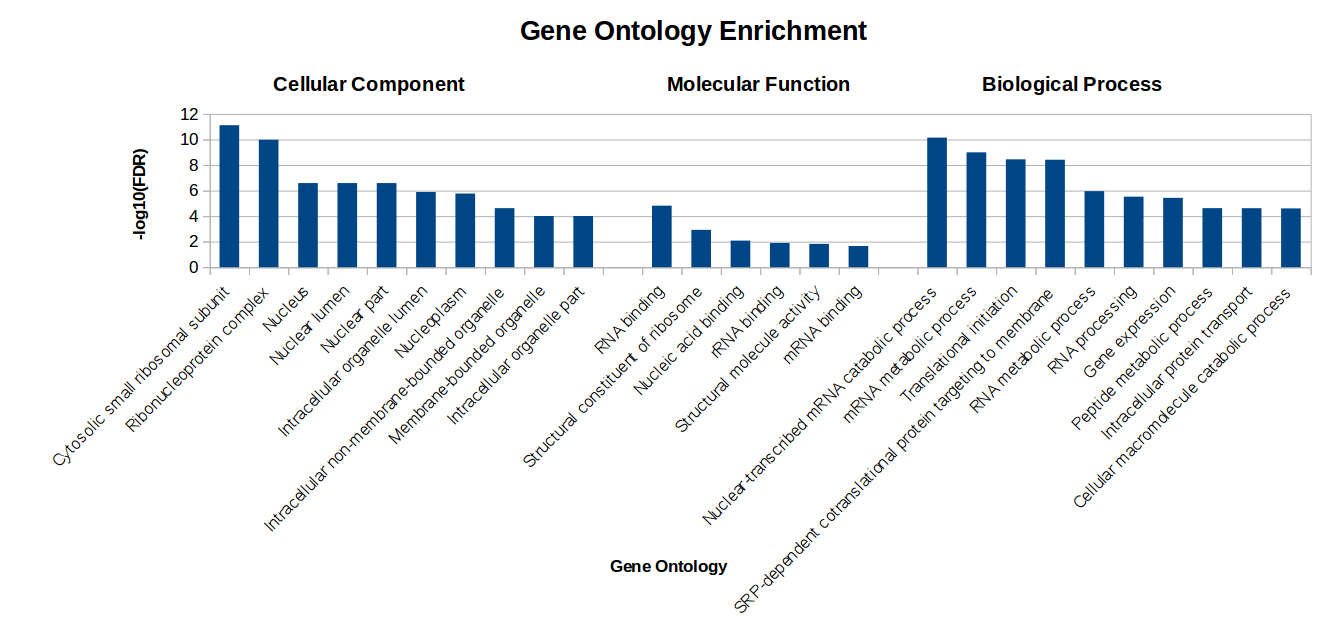

Supplement: Supplementary file 5 — Figure S2. Gene ontology enrichment graph. (TIFF 206 kb) [file 12864_2019_5794_MOESM5_ESM.tiff]

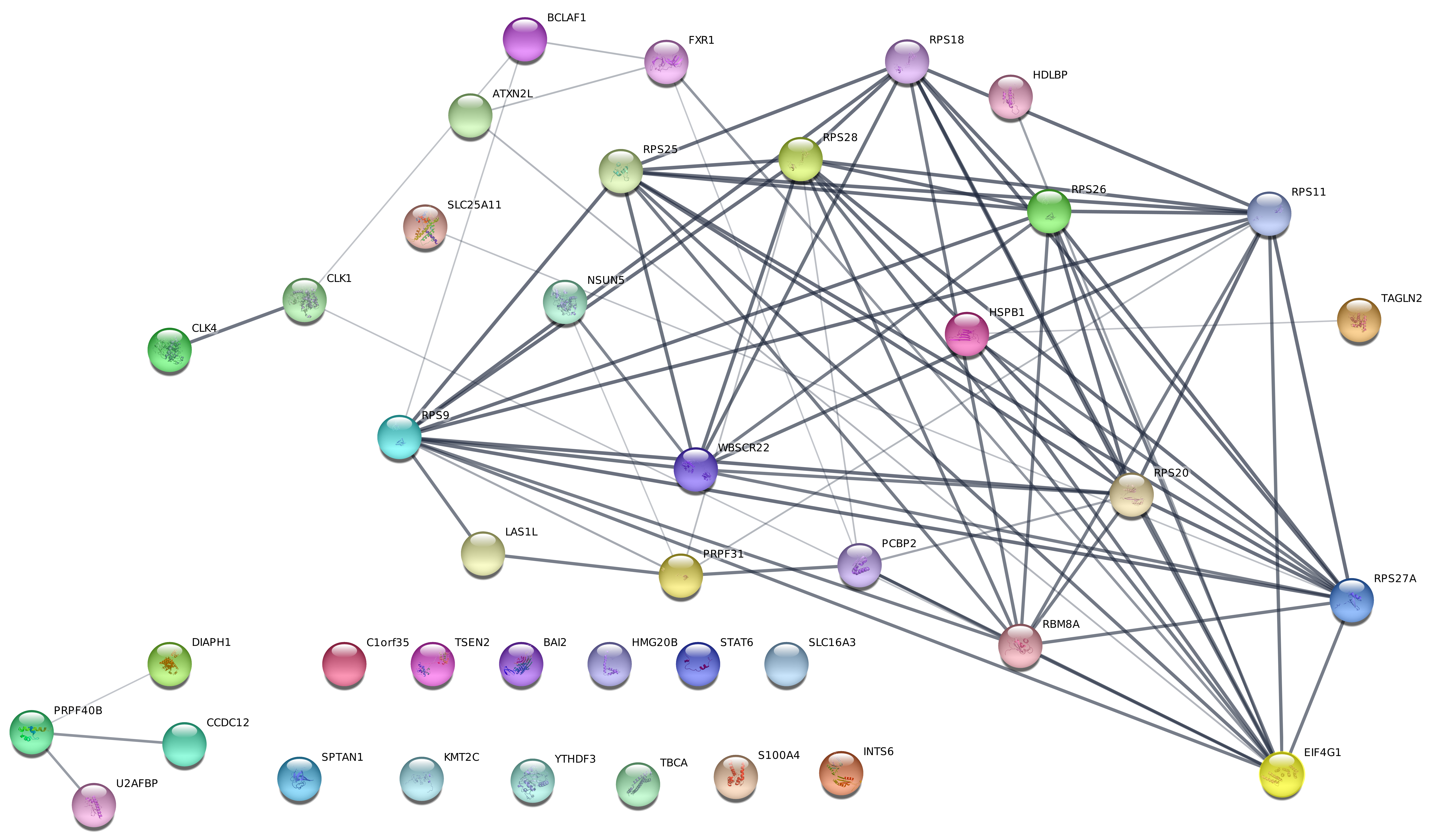

Supplement: Supplementary file 7 — Figure S3. Network analysis of all the 40 common genes. (TIFF 3427 kb) [file 12864_2019_5794_MOESM7_ESM.tiff]
