# Supplementary material for: Events of alternative splicing in head and neck cancer via RNA sequencing – an update
Source: BMC Genomics. 2019 Jun 3;20:442. doi: 10.1186/s12864-019-5794-y (PMC6545735; doi:10.1186/s12864-019-5794-y)
Supplement: Supplementary file 3 — Criteria of screening studies. (DOC 48 kb) [file 12864_2019_5794_MOESM3_ESM.doc]

**Additional file 3**

**Criteria of screening studies**

The screening of studies was performed on PubMed, and Web of Science databases on the 19^th^ of September, 2017. “Shafer’s Textbook of Oral Pathology 7^th^ Edition” (https://drive.google.com/file/d/0B1yiBOBAXbBpeWFCdjkyUVFLck0/view?ts=594127d1) was consulted for making synonym where different terms on head and neck cancer were used. In addition, Google (1^st^ November 2017) was also consulted for including studies not captured through PubMed, and Web of Science search. A total of 323 studies were obtained from PubMed, 887 studies from Web of Science and 3 from Google search. None of the studies duplicated from databases, hence total of 1213 studies were obtained during a comprehensive search (Table S1). None of the studies matched to our topic of interest. The details of phrases used are given below:

**Phrases used for screening the records**

**Phrase in PubMed**

(("mouth neoplasms"[MeSH Terms] OR ("mouth"[All Fields] AND "neoplasms"[All Fields]) OR "mouth neoplasms"[All Fields] OR ("oral"[All Fields] AND "cancer"[All Fields]) OR "oral cancer"[All Fields]) OR OSCC[All Fields] OR (("mouth"[MeSH Terms] OR "mouth"[All Fields] OR "oral"[All Fields]) AND ("carcinoma, squamous cell"[MeSH Terms] OR ("carcinoma"[All Fields] AND "squamous"[All Fields] AND "cell"[All Fields]) OR "squamous cell carcinoma"[All Fields] OR ("squamous"[All Fields] AND "cell"[All Fields] AND "carcinoma"[All Fields]))) OR (("carcinoma, squamous cell"[MeSH Terms] OR ("carcinoma"[All Fields] AND "squamous"[All Fields] AND "cell"[All Fields]) OR "squamous cell carcinoma"[All Fields] OR ("squamous"[All Fields] AND "cell"[All Fields] AND "carcinoma"[All Fields])) AND ("mouth"[MeSH Terms] OR "mouth"[All Fields] OR ("oral"[All Fields] AND "cavity"[All Fields]) OR "oral cavity"[All Fields])) OR (("carcinoma, squamous cell"[MeSH Terms] OR ("carcinoma"[All Fields] AND "squamous"[All Fields] AND "cell"[All Fields]) OR "squamous cell carcinoma"[All Fields] OR ("squamous"[All Fields] AND "cell"[All Fields] AND "carcinoma"[All Fields])) AND ("lip"[MeSH Terms] OR "lip"[All Fields])) OR (("carcinoma, squamous cell"[MeSH Terms] OR ("carcinoma"[All Fields] AND "squamous"[All Fields] AND "cell"[All Fields]) OR "squamous cell carcinoma"[All Fields] OR ("squamous"[All Fields] AND "cell"[All Fields] AND "carcinoma"[All Fields])) AND ("mouth mucosa"[MeSH Terms] OR ("mouth"[All Fields] AND "mucosa"[All Fields]) OR "mouth mucosa"[All Fields] OR ("buccal"[All Fields] AND "mucosa"[All Fields]) OR "buccal mucosa"[All Fields])) OR (("carcinoma, squamous cell"[MeSH Terms] OR ("carcinoma"[All Fields] AND "squamous"[All Fields] AND "cell"[All Fields]) OR "squamous cell carcinoma"[All Fields] OR ("squamous"[All Fields] AND "cell"[All Fields] AND "carcinoma"[All Fields])) AND ("tongue"[MeSH Terms] OR "tongue"[All Fields])) OR ("carcinoma, basal cell"[MeSH Terms] OR ("carcinoma"[All Fields] AND "basal"[All Fields] AND "cell"[All Fields]) OR "basal cell carcinoma"[All Fields] OR ("basal"[All Fields] AND "cell"[All Fields] AND "carcinoma"[All Fields])) OR (("carcinoma"[MeSH Terms] OR "carcinoma"[All Fields]) AND ("mouth floor"[MeSH Terms] OR ("mouth"[All Fields] AND "floor"[All Fields]) OR "mouth floor"[All Fields] OR ("floor"[All Fields] AND "mouth"[All Fields]) OR "floor of the mouth"[All Fields])) OR (("carcinoma"[MeSH Terms] OR "carcinoma"[All Fields]) AND ("palate"[MeSH Terms] OR "palate"[All Fields])) OR (("carcinoma"[MeSH Terms] OR "carcinoma"[All Fields]) AND ("gingiva"[MeSH Terms] OR "gingiva"[All Fields] OR "gingival"[All Fields])) OR (("carcinoma"[MeSH Terms] OR "carcinoma"[All Fields]) AND ("maxillary sinus"[MeSH Terms] OR ("maxillary"[All Fields] AND "sinus"[All Fields]) OR "maxillary sinus"[All Fields])) OR ("carcinoma, verrucous"[MeSH Terms] OR ("carcinoma"[All Fields] AND "verrucous"[All Fields]) OR "verrucous carcinoma"[All Fields] OR ("verrucous"[All Fields] AND "carcinoma"[All Fields])) OR ("carcinoma"[MeSH Terms] OR "carcinoma"[All Fields] OR ("polypoid"[All Fields] AND "squamous"[All Fields] AND "cell"[All Fields] AND "carcinoma"[All Fields]) OR "polypoid squamous cell carcinoma"[All Fields]) OR (Lane[All Fields] AND ("tumour"[All Fields] OR "neoplasms"[MeSH Terms] OR "neoplasms"[All Fields] OR "tumor"[All Fields])) OR (("adenoids"[MeSH Terms] OR "adenoids"[All Fields] OR "adenoid"[All Fields]) AND ("carcinoma, squamous cell"[MeSH Terms] OR ("carcinoma"[All Fields] AND "squamous"[All Fields] AND "cell"[All Fields]) OR "squamous cell carcinoma"[All Fields] OR ("squamous"[All Fields] AND "cell"[All Fields] AND "carcinoma"[All Fields]))) OR adenoacanthoma[All Fields] OR (basaloid[All Fields] AND ("carcinoma, squamous cell"[MeSH Terms] OR ("carcinoma"[All Fields] AND "squamous"[All Fields] AND "cell"[All Fields]) OR "squamous cell carcinoma"[All Fields] OR ("squamous"[All Fields] AND "cell"[All Fields] AND "carcinoma"[All Fields]))) OR ("carcinoma, adenosquamous"[MeSH Terms] OR ("carcinoma"[All Fields] AND "adenosquamous"[All Fields]) OR "adenosquamous carcinoma"[All Fields] OR ("adenosquamous"[All Fields] AND "carcinoma"[All Fields])) OR lymphoepithelioma[All Fields] OR ("carcinoma, mucoepidermoid"[MeSH Terms] OR ("carcinoma"[All Fields] AND "mucoepidermoid"[All Fields]) OR "mucoepidermoid carcinoma"[All Fields] OR ("mucoepidermoid"[All Fields] AND "carcinoma"[All Fields])) OR ("carcinoma, adenoid cystic"[MeSH Terms] OR ("carcinoma"[All Fields] AND "adenoid"[All Fields] AND "cystic"[All Fields]) OR "adenoid cystic carcinoma"[All Fields] OR ("adenoid"[All Fields] AND "cystic"[All Fields] AND "carcinoma"[All Fields])) OR ("carcinoma, adenoid cystic"[MeSH Terms] OR ("carcinoma"[All Fields] AND "adenoid"[All Fields] AND "cystic"[All Fields]) OR "adenoid cystic carcinoma"[All Fields] OR ("adenocystic"[All Fields] AND "carcinoma"[All Fields]) OR "adenocystic carcinoma"[All Fields]) OR ("cystadenocarcinoma, papillary"[MeSH Terms] OR ("cystadenocarcinoma"[All Fields] AND "papillary"[All Fields]) OR "papillary cystadenocarcinoma"[All Fields] OR ("papillary"[All Fields] AND "cystadenocarcinoma"[All Fields])) OR (("salivary ducts"[MeSH Terms] OR ("salivary"[All Fields] AND "ducts"[All Fields]) OR "salivary ducts"[All Fields] OR ("salivary"[All Fields] AND "duct"[All Fields]) OR "salivary duct"[All Fields]) AND ("carcinoma"[MeSH Terms] OR "carcinoma"[All Fields])) OR (("salivary ducts"[MeSH Terms] OR ("salivary"[All Fields] AND "ducts"[All Fields]) OR "salivary ducts"[All Fields] OR ("salivary"[All Fields] AND "duct"[All Fields]) OR "salivary duct"[All Fields]) AND ("adenocarcinoma"[MeSH Terms] OR "adenocarcinoma"[All Fields])) OR (ameloblastic[All Fields] AND ("carcinoma"[MeSH Terms] OR "carcinoma"[All Fields])) OR (("erythrocyte membrane"[MeSH Terms] OR ("erythrocyte"[All Fields] AND "membrane"[All Fields]) OR "erythrocyte membrane"[All Fields] OR "ghost"[All Fields]) AND ("cells"[MeSH Terms] OR "cells"[All Fields] OR "cell"[All Fields]) AND odontogenic[All Fields] AND ("carcinoma"[MeSH Terms] OR "carcinoma"[All Fields])) OR (("margins of excision"[MeSH Terms] OR ("margins"[All Fields] AND "excision"[All Fields]) OR "margins of excision"[All Fields] OR "clear"[All Fields]) AND ("cells"[MeSH Terms] OR "cells"[All Fields] OR "cell"[All Fields]) AND odontogenic[All Fields] AND ("carcinoma"[MeSH Terms] OR "carcinoma"[All Fields])) OR (("neoplasms"[MeSH Terms] OR "neoplasms"[All Fields] OR "cancers"[All Fields]) AND ("nasal cavity"[MeSH Terms] OR ("nasal"[All Fields] AND "cavity"[All Fields]) OR "nasal cavity"[All Fields])) OR ("Nasopharyngeal carcinoma"[Supplementary Concept] OR "Nasopharyngeal carcinoma"[All Fields] OR "nasopharyngeal carcinoma"[All Fields]) OR (("african continental ancestry group"[MeSH Terms] OR ("african"[All Fields] AND "continental"[All Fields] AND "ancestry"[All Fields] AND "group"[All Fields]) OR "african continental ancestry group"[All Fields] OR "african"[All Fields]) AND ("jaw"[MeSH Terms] OR "jaw"[All Fields]) AND ("lymphoma"[MeSH Terms] OR "lymphoma"[All Fields])) OR ("oropharyngeal neoplasms"[MeSH Terms] OR ("oropharyngeal"[All Fields] AND "neoplasms"[All Fields]) OR "oropharyngeal neoplasms"[All Fields] OR ("oropharyngeal"[All Fields] AND "cancer"[All Fields]) OR "oropharyngeal cancer"[All Fields]) OR (ameloblastic[All Fields] AND ("fibrosarcoma"[MeSH Terms] OR "fibrosarcoma"[All Fields])) OR ("laryngeal neoplasms"[MeSH Terms] OR ("laryngeal"[All Fields] AND "neoplasms"[All Fields]) OR "laryngeal neoplasms"[All Fields] OR ("laryngeal"[All Fields] AND "cancer"[All Fields]) OR "laryngeal cancer"[All Fields]) OR (("larynx"[MeSH Terms] OR "larynx"[All Fields] OR "laryngeal"[All Fields]) AND ("carcinoma, squamous cell"[MeSH Terms] OR ("carcinoma"[All Fields] AND "squamous"[All Fields] AND "cell"[All Fields]) OR "squamous cell carcinoma"[All Fields] OR ("squamous"[All Fields] AND "cell"[All Fields] AND "carcinoma"[All Fields]))) OR ("hypopharyngeal neoplasms"[MeSH Terms] OR ("hypopharyngeal"[All Fields] AND "neoplasms"[All Fields]) OR "hypopharyngeal neoplasms"[All Fields] OR ("hypopharyngeal"[All Fields] AND "cancer"[All Fields]) OR "hypopharyngeal cancer"[All Fields]) OR ("head and neck neoplasms"[MeSH Terms] OR ("head"[All Fields] AND "neck"[All Fields] AND "neoplasms"[All Fields]) OR "head and neck neoplasms"[All Fields] OR ("head"[All Fields] AND "neck"[All Fields] AND "cancer"[All Fields]) OR "head and neck cancer"[All Fields]) OR (("Head Neck"[Journal] OR ("head"[All Fields] AND "and"[All Fields] AND "neck"[All Fields]) OR "head and neck"[All Fields]) AND ("carcinoma, squamous cell"[MeSH Terms] OR ("carcinoma"[All Fields] AND "squamous"[All Fields] AND "cell"[All Fields]) OR "squamous cell carcinoma"[All Fields] OR ("squamous"[All Fields] AND "cell"[All Fields] AND "carcinoma"[All Fields]))) OR HNSCC[All Fields] OR (HN[All Fields] AND ("neoplasms"[MeSH Terms] OR "neoplasms"[All Fields] OR "cancer"[All Fields]))) AND (("alternative splicing"[MeSH Terms] OR ("alternative"[All Fields] AND "splicing"[All Fields]) OR "alternative splicing"[All Fields] OR ("alternate"[All Fields] AND "splicing"[All Fields]) OR "alternate splicing"[All Fields]) OR (mutually[All Fields] AND exclusive[All Fields] AND ("exons"[MeSH Terms] OR "exons"[All Fields] OR "exon"[All Fields])) OR (retained[All Fields] AND ("introns"[MeSH Terms] OR "introns"[All Fields])) OR (("introns"[MeSH Terms] OR "introns"[All Fields] OR "intron"[All Fields]) AND ("retention (psychology)"[MeSH Terms] OR ("retention"[All Fields] AND "(psychology)"[All Fields]) OR "retention (psychology)"[All Fields] OR "retention"[All Fields])) OR (cassette[All Fields] AND ("exons"[MeSH Terms] OR "exons"[All Fields] OR "exon"[All Fields]) AND skipping[All Fields]) OR (alternative[All Fields] AND 5'and[All Fields] AND ("rna splice sites"[MeSH Terms] OR ("rna"[All Fields] AND "splice"[All Fields] AND "sites"[All Fields]) OR "rna splice sites"[All Fields] OR ("3'"[All Fields] AND "splice"[All Fields] AND "site"[All Fields]) OR "3' splice site"[All Fields]))) AND ((("rna"[MeSH Terms] OR "rna"[All Fields]) AND Seq[All Fields]) OR ("sequence analysis, rna"[MeSH Terms] OR ("sequence"[All Fields] AND "analysis"[All Fields] AND "rna"[All Fields]) OR "rna sequence analysis"[All Fields] OR ("rna"[All Fields] AND "sequencing"[All Fields]) OR "rna sequencing"[All Fields])) AND ("humans"[MeSH Terms] OR "humans"[All Fields]).

1. Phrase in Web of Science

TITLE: (oral cancer OR OSCC OR oral squamous cell carcinoma OR squamous cell carcinoma of the oral cavity) ORTITLE: (squamous cell carcinoma of lip OR squamous cell carcinoma of buccal mucosa OR squamous cell carcinoma of tongue) OR TITLE:(basal cell carcinoma OR carcinoma of floor of the mouth OR carcinoma of palate OR carcinoma of gingival OR carcinoma of maxillary sinus) ORTITLE: (verrucous carcinoma OR polypoid squamous cell carcinoma OR Lane tumor OR adenoid squamous cell carcinoma OR adenoacanthoma) OR TITLE: (basaloid squamous cell carcinoma OR adenosquamous carcinoma OR lymphoepithelioma OR mucoepidermoid carcinoma OR adenoid cystic carcinoma OR adenocystic carcinoma) OR TITLE:(papillary cystadenocarcinoma OR salivary duct carcinoma OR salivary duct adenocarcinoma OR ameloblastic carcinoma OR ghost cell odontogenic carcinoma) OR TITLE:(clear cell odontogenic carcinoma OR cancers of the nasal cavity OR nasopharyngeal carcinoma OR african jaw lymphoma) OR TITLE:(ameloblastic fibrosarcoma OR laryngeal cancer) OR TITLE:(lypopharyngeal cancer) OR TITLE:(HN cancer) OR TITLE: (HNSCC) ORTITLE: (head and neck squamous cell carcinoma) OR TITLE: (head and neck cancer) OR TITLE: (laryngeal squamous cell carcinoma) OR TITLE:(oropharyngeal cancer) AND TOPIC:(alternate splicing OR mutually exclusive exon OR retained introns OR intron retention OR cassette exon skipping OR alternative 5 and 3 splice site) AND TOPIC: (RNA Seq OR RNA sequencing) AND TOPIC: (humans)

Refined by: PUBLICATION YEARS: ( 2016 OR 2015 OR 2014 OR 2013 OR 2012 ) AND DOCUMENT TYPES: ( ARTICLE OR REVIEW ) AND Open Access: ( YES ) AND WEB OF SCIENCE CATEGORIES: ( ONCOLOGY OR BIOTECHNOLOGY APPLIED MICROBIOLOGY OR MICROBIOLOGY OR PATHOLOGY OR CELL BIOLOGY OR OTORHINOLARYNGOLOGY OR DENTISTRY ORAL SURGERY MEDICINE OR GENETICS HEREDITY OR BIOLOGY OR MEDICAL INFORMATICS ) AND WEB OF SCIENCE CATEGORIES: ( ONCOLOGY OR BIOLOGY OR PATHOLOGY OR INFECTIOUS DISEASES OR CELL BIOLOGY OR HEMATOLOGY OR OTORHINOLARYNGOLOGY OR MEDICAL INFORMATICS OR CELL TISSUE ENGINEERING OR BIOCHEMISTRY MOLECULAR BIOLOGY OR BIOCHEMICAL RESEARCH METHODS OR DENTISTRY ORAL SURGERY MEDICINE OR GENETICS HEREDITY ) AND DOCUMENT TYPES: ( ARTICLE OR REVIEW ) AND WEB OF SCIENCE CATEGORIES: ( ONCOLOGY ) AND LANGUAGES: ( ENGLISH ) AND RESEARCH AREAS: ( ONCOLOGY ) ANDLANGUAGES: ( ENGLISH )

Timespan: 2010-2017. Indexes: SCI-EXPANDED
