# Supplementary material for: Events of alternative splicing in head and neck cancer via RNA sequencing – an update
Source: BMC Genomics. 2019 Jun 3;20:442. doi: 10.1186/s12864-019-5794-y (PMC6545735; doi:10.1186/s12864-019-5794-y)
Supplement: Supplementary file 8 — Table S2. Summary of the alternative splicing detection softwares and their implementation in cancer research. (DOC 139 kb) [file 12864_2019_5794_MOESM8_ESM.doc]

**Table S2** Summary of the alternative splicing detection softwares and their implementation in cancer research.

| **S. No** | **Software** | **References** | **Year** | **URL** | **Implementation in cancer research** |
| --- | --- | --- | --- | --- | --- |
|
| 1 | SplAdder | Kahles *et al*. [1] | 2016 | https://github.com/ratschlab/spladder | Glioblastoma [2] |
| 2 | Asprofile | Florea *et al*. [3] | 2013 | http://ccb.jhu.edu/software/ASprofile/ | adipose tissue, brain tissue, breast tissue, colon tissue, heart tissue, kidney tissue, liver tissue, lung tissue, lymph tissue, ovary tissue, prostate tissue, skeletal muscle tissue, testes tissue, thyroid tissue and white blood cells [3], breast cancer [4] |
| 3 | JuncBASE | Brooks *et al*. [5] | 2012 | https://github.com/anbrooks/juncBASE | chronic lymphocytic leukemia [6] |
| 4 | MATS | Shen *et al*. [7] | 2012 | http://rnaseq-mats.sourceforge.net/ | breast cancer [4] |
| 5 | jSplice | Christinat *et al*. [8] | 2016 | http://www.mhs.biol.ethz.ch/research/krek/jsplice | renal carcinoma cells [9] |
| 6 | DEXSeq | Li *et al*. [10] | 2015 | http://bioconductor.org/packages/release/bioc/html/DEXSeq.html | brain tissue, heart tissue, liver tissue, and muscle tissue [11] |
| 7 | DSGseq | Wang *et al*. [12] | 2013 | http://bioinfo.au.tsinghua.edu.cn/software/DSGseq/ | prostate cancer [13] |
| 8 | DiffSplice | Hu *et al*. [14] | 2013 | http://www.netlab.uky.edu/p/bioinfo/DiffSplice | human bronchial cells and breast cancer [14] |

| **S. No** | **Software** | **References** | **Year** | **URL** | **Implementation in cancer research** |
| --- | --- | --- | --- | --- | --- |
|
| 9 | SGSeq | Goldstein *et al*. [15] | 2016 | http://bioconductor.org/packages/release/bioc/html/SGSeq.html | normal human tissue samples [15] |
| 10 | ESFinder | Bai *et al*. [16] | 2015 | http://www.mybiosoftware.com/esfinder-exon-skipping-event-identification-from-rna-seq-data.html | human skeletal muscle and brain [16] |
| 11 | GESS | Ye *et al*. [17] | 2014 | http://compbio.uthscsa.edu/GESS_Web/ | prostate cancer [17] |
| 12 | Alt Event Finder | Zhou *et al*. [18] | 2012 | http://compbio.iupui.edu/group/6/pages/alteventfinder | human primary hepatocytes [18] |

**References**

1. Kahles A, Ong CS, Zhong Y, Rätsch G. SplAdder: identification, quantification and testing of alternative splicing events from RNA-Seq data. Bioinformatics [Internet]. 2016;32:1840–7. Available from: http://www.ncbi.nlm.nih.gov/pubmed/26873928

2. Esteve-Codina A, Arpi O, Martinez-García M, Pineda E, Mallo M, Gut M, et al. A Comparison of RNA-Seq Results from Paired Formalin-Fixed Paraffin-Embedded and Fresh-Frozen Glioblastoma Tissue Samples. PLoS One [Internet]. 2017;12:e0170632. Available from: http://www.ncbi.nlm.nih.gov/pubmed/28122052

3. Florea L, Song L, Salzberg SL. Thousands of exon skipping events differentiate among splicing patterns in sixteen human tissues. F1000Research [Internet]. 2013;2:188. Available from: http://www.ncbi.nlm.nih.gov/pubmed/24555089

4. Eswaran J, Horvath A, Godbole S, Reddy SD, Mudvari P, Ohshiro K, et al. RNA sequencing of cancer reveals novel splicing alterations. Sci Rep [Internet]. 2013;3:1689. Available from: http://www.ncbi.nlm.nih.gov/pubmed/23604310

5. Brooks AN, Yang L, Duff MO, Hansen KD, Park JW, Dudoit S, et al. Conservation of an RNA regulatory map between Drosophila and mammals. Genome Res [Internet]. 2011;21:193–202. Available from: http://www.ncbi.nlm.nih.gov/pubmed/20921232

6. Wang L, Brooks AN, Fan J, Wan Y, Gambe R, Li S, et al. Transcriptomic Characterization of SF3B1 Mutation Reveals Its Pleiotropic Effects in Chronic Lymphocytic Leukemia. Cancer Cell [Internet]. 2016;30:750–63. Available from: http://www.ncbi.nlm.nih.gov/pubmed/27818134

7. Shen S, Park JW, Huang J, Dittmar KA, Lu Z, Zhou Q, et al. MATS: a Bayesian framework for flexible detection of differential alternative splicing from RNA-Seq data. Nucleic Acids Res [Internet]. 2012;40:e61. Available from: http://www.ncbi.nlm.nih.gov/pubmed/22266656

8. Christinat Y, Pawłowski R, Krek W. jSplice: a high-performance method for accurate prediction of alternative splicing events and its application to large-scale renal cancer transcriptome data. Bioinformatics [Internet]. 2016;32:2111–9. Available from: http://www.ncbi.nlm.nih.gov/pubmed/27153587

9. Bai Y, Ji S, Wang Y. IRcall and IRclassifier: two methods for flexible detection of intron retention events from RNA-Seq data. BMC Genomics [Internet]. 2015;16 Suppl 2:S9. Available from: http://www.ncbi.nlm.nih.gov/pubmed/25707295

10. Li Y, Rao X, Mattox WW, Amos CI, Liu B. RNA-Seq Analysis of Differential Splice Junction Usage and Intron Retentions by DEXSeq. PLoS One [Internet]. 2015;10:e0136653. Available from: http://www.ncbi.nlm.nih.gov/pubmed/26327458

11. Badr E, ElHefnawi M, Heath LS. Computational Identification of Tissue-Specific Splicing Regulatory Elements in Human Genes from RNA-Seq Data. PLoS One [Internet]. 2016;11:e0166978. Available from: http://www.ncbi.nlm.nih.gov/pubmed/27861625

12. Wang W, Qin Z, Feng Z, Wang X, Zhang X. Identifying differentially spliced genes from two groups of RNA-seq samples. Gene [Internet]. 2013;518:164–70. Available from: http://www.ncbi.nlm.nih.gov/pubmed/23228854

13. Feng Huijuan, Li Tingting ZX. Characterization of kinase gene expression and splicing profile in prostate cancer with RNA-Seq data. 2016; Available from: https://www.biorxiv.org/content/early/2016/06/29/061085

14. Hu Y, Huang Y, Du Y, Orellana CF, Singh D, Johnson AR, et al. DiffSplice: the genome-wide detection of differential splicing events with RNA-seq. Nucleic Acids Res [Internet]. 2013;41:e39. Available from: http://www.ncbi.nlm.nih.gov/pubmed/23155066

15. Goldstein LD, Cao Y, Pau G, Lawrence M, Wu TD, Seshagiri S, et al. Prediction and Quantification of Splice Events from RNA-Seq Data. PLoS One [Internet]. 2016;11:e0156132. Available from: http://www.ncbi.nlm.nih.gov/pubmed/27218464

16. Bai Y, Ji S, Jiang Q, Wang Y. Identification Exon Skipping Events From High-Throughput RNA Sequencing Data. IEEE Trans Nanobioscience [Internet]. 2015;14:562–9. Available from: http://www.ncbi.nlm.nih.gov/pubmed/25935040

17. Wang J, Ye Z, Huang TH, Shi H, Jin VX. Computational Methods and Correlation of Exon-skipping Events with Splicing, Transcription, and Epigenetic Factors. Methods Mol Biol [Internet]. 2017;1513:163–70. Available from: http://www.ncbi.nlm.nih.gov/pubmed/27807836

18. Zhou A, Breese MR, Hao Y, Edenberg HJ, Li L, Skaar TC, et al. Alt Event Finder: a tool for extracting alternative splicing events from RNA-seq data. BMC Genomics [Internet]. 2012;13 Suppl 8:S10. Available from: http://www.ncbi.nlm.nih.gov/pubmed/23281921
